# An intermolecular-split G-quadruplex DNAzyme sensor for dengue virus detection†

**DOI:** 10.1039/d0ra05439a

**Published:** 2020-09-07

**Authors:** Jeunice Ida, Akinori Kuzuya, Yee Siew Choong, Theam Soon Lim

**Affiliations:** Institute for Research in Molecular Medicine, Universiti Sains Malaysia 11800 Penang Malaysia theamsoon@usm.my +60-4-653-4803 +60-4-653-4852; Department of Chemistry and Materials Engineering, Kansai University 3-3-35 Yamate, Suita Osaka 564-8680 Japan; Analytical Biochemistry Research Centre, Universiti Sains Malaysia 11800 Penang Malaysia

## Abstract

Nucleic acids have special ability to organize themselves into various non-canonical structures, including a four-stranded DNA structure termed G-quadruplex (G4) that has been utilized for diagnostic and therapeutic applications. Herein, we report the ability of G4 to distinguish dengue virus (DENV) based on its serotypes (DENV-1, DENV-2, DENV-3 and DENV-4) using a split G4-hemin DNAzyme configuration. In this system, two separate G-rich oligonucleotides are brought together upon target DNA strand hybridization to form a three-way junction architecture, allowing the formation of a G4 structure. The G4 formation in complexation with hemin can thus provide a signal readout by generating a DNAzyme that is able to catalyze H_2_O_2_-mediated oxidation of 2,2′-azino-bis(3-ethylbenzothiazoline-6-sulfonic acid) diammonium salt (ABTS). This results in a change of color providing a sensing platform for the colorimetric detection of DENV. In our approach, betaine and dimethyl sulfoxide were utilized for better G4 generation by enhancing the target-probe hybridization. In addition to this serotype-specific assay, a multi-probe cocktail assay, which is an all-in-one assay was also examined for DENV detection. The system highlights the potential of split G-quadruplex configurations for the development of DNA-based detection and serotyping systems in the future.

## Abstract 2

Application of split G-quadruplex as DNAzyme reporter system for DNA sensing.

## Graphical abstract

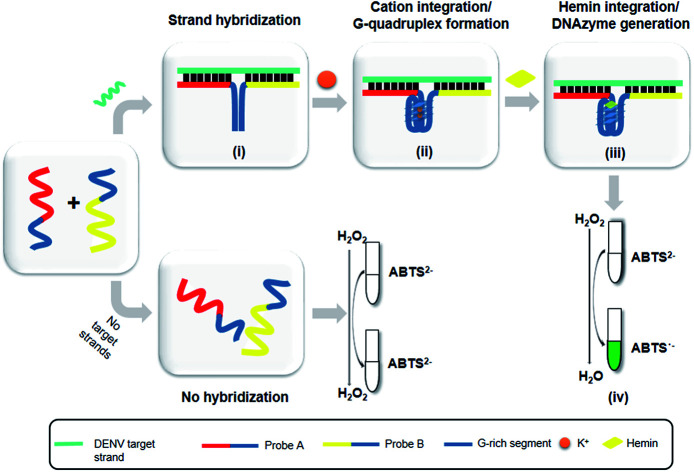

## Introduction

Serving as a genetic information storage system for all living organisms as well as many viruses, deoxyribonucleic acid (DNA) has allowed scientists to identify key organisms based on sequences that are unique to specific organism.^1^ Medical diagnostics can take advantage of these useful characteristics as the continued threat of various organisms and viruses against humans has become a serious public concern.^2^ DNA-based detection systems are attractive alternatives for the diagnosis of infection, where the unique DNA sequences of target organisms or viruses are identified. Generally, for an *in vitro* setup, the detection of any biological information requires several key components. This includes unique segments of target organisms, specific target binders to capture the segments, and last but not least, a reporter system that is capable of distinguishing the presence of target organisms. An attractive advantage of DNA-based sensors is that this platform is highly dependent on the meeting and strong interaction of the target DNA and DNA anchor/probe. Due to this complementary type approach, this platform is highly specific.^3^ However, the choice of a suitable detection platform is a key factor in DNA sensing. In many DNA-based detection systems, chemical modification and labelling, whether coupled with fluorescent dyes, dye pigments, nanoparticles or enzymes, can yield different types of readouts. A good yet less costly alternative to the use of modified oligonucleotides is the utilization of oligonucleotide itself to act as a reporter for the generation of visible signals. This is plausible with the employment of a higher order type of DNA structure termed as G-quadruplex.

The versatility of G4 has captured growing attention from different research groups for diagnostic assay development.^4–7^ This has helped shape the way we perceive DNA structures today and has allowed researchers to construct functional structures, expanding the presumed traditional roles of DNA. Consequently, arising from one, two or four guanine (G)-rich strand(s), G-tracts linked by loop sequences are formed. Association of a G base through Hoogsteen hydrogen bonding with another three G bases from neighbouring G-tracts form a square planar structure, termed a G-quartet or G-tetrad. The formed G-quartets stack on top of one another to form a G4 structure in the presence of stabilizing cations such as potassium and sodium. G4 is highly polymorphic and exhibits a unique geometry; this allows G4 to recognize and detect small molecules, metal ions, DNA, enzyme activity and proteins through multiple modes of binding.^8^ Interestingly, the binding of hemin (iron(iii)-protoporphyrin IX) to G4 can be observed visually, as the base-specific steric configuration of G4 allows the stacked G-quartets to bind to it. In this way, binding regulates an enzymatic-like function that mimics peroxidase activity, generating a DNAzyme that catalyses H_2_O_2_-oxidation of 2,2′-azinobis(3-ethylbenzothiazoline-6-sulfonic acid) (ABTS^2−^) to ABTS^·-^. This in turn generates a colorimetric change from a colourless solution to a visible green product.^9,10^

Herein, the proposed assay utilizes split G4 structures as a reporter system for dengue DNA sensing. The capability of this approach to distinguish different sequences which share low degrees of variation was tested. This approach has been shown to be sensitive towards single-nucleotide mismatches,^5^ which have provoked consideration of its serotyping ability. Therefore, dengue virus (DENV), which consists of four serotypes, was utilized as a suitable model target to develop a split G4-based DNA sensor. In this study, the structure of the G-rich sequences was split into two parts, which then functioned as binary probes. We demonstrated a simple DNA-based system for DENV serotyping and detection which takes advantage of DNAzymes generated from split G4-hemin complexes. This formation originated from single-stranded DNA (ssDNA) molecules that can recognize and anneal to their complementary strands in a sample with stable hydrogen bond formation. These ssDNA molecules are made to carry a protruding G-rich sequence, which subsequently forms a G4 complex once the protruding strands are brought together.

This splitting approach is thought to allow higher selectivity and specificity.^11^ It may also afford higher flexibility in that it permits G-rich segments to be integrated into more than one pattern, yielding different signal-to-background ratios. The total number of guanine bases needed to from a split G4 is often twelve.^12^ Hence, in this work, the commonly used guanine number was preserved, and the whole probe sequences were initially split into halves (symmetrical splitting), with the probes containing six G bases on each side. Asymmetrical splitting was attempted in the conditions where symmetrical splitting did not favour the detection assay. This was done by changing the ratio of the number of guanine bases from 6 : 6 to 8 : 4 and eventually 10 : 2.

To enhance the activity of the assay, the influence of enhancing agents such as betaine and dimethyl sulfoxide was also investigated. In contrast to other DNA-based sensors, such as molecular beacons, this assay does not involve any chemical modification or labelling. Therefore, it is less laborious and inexpensive to construct probes for this detection assay. In addition to being inexpensive, this system provides a simpler methodology, omitting the need for trained expertise to conduct the assay. Due to its simple operational method and lack of requirement of sophisticated instruments, it would be highly convenient for small-scale clinical laboratories, especially in developing regions, where dengue is endemic.

DENV itself has become a serious public concern, as 390 million dengue virus infections are reported every year in over 100 countries. Dengue is a mosquito-borne viral disease caused by dengue virus (DENV), which is a member of the *Flavivirus* genus. It is transmitted by Aedes mosquitoes, mainly *A. aegypti* and, to a lesser extent, *A. albopictus*. Even with advancements in medical sciences, dengue remains a major health challenge to the global community.^13,14^ Infections can be caused by any of the four serotypes of DENV (DENV-1, DENV-2, DENV-3 and DENV-4) and can trigger unique immune responses in patients. Infected persons develop a lifelong immunity to the infecting serotype; however, they are exposed to a higher risk of severe dengue shock syndrome if they are infected with any of the three other serotypes.^15,16^ Therefore, DENV serotyping is vital, especially during transmission seasons, as a measure for disease epidemiology. Also, DENVs share the same symptoms with other flaviviruses, such as Zika virus (ZIKV).^17,18^ With no pathognomonic characteristics that reliably discriminate one DENV serotype from another and from other febrile diseases, confirmation through laboratory diagnosis is crucial.

To date, several dengue diagnosis platforms have been investigated and developed in search of an ideal identification method. The main platforms include NS1 antigen detection,^19–21^ IgM and IgG antibody detection^22–31^ and various DNA amplification methods.^32–37^ While many different DENV detection methods are available for DENV identification, no single methodology has been proved to meet the requirements of being sensitive, specific, rapid and inexpensive at the same time.^38^ This scenario calls for alternative detection techniques that can fill this gap for the diagnosis of DENV.

## Materials and methods

### Reagents and apparatus

The target and probe sequences used in this assay were synthesized by Integrated DNA Technology (Iowa, US) and are listed in Table 1. The DENV sequence data of each serotype prototype strain were retrieved from GenBank: DENV-1 (MG877557.1), DENV-2 (KY977454.1), DENV-3 (MF004386.1) and DENV-4 (MF004387.1) (the numbers shown in parentheses are the accession numbers). Sequences representative of all four DENV serotypes were aligned using Clustal Omega. Dimethyl sulfoxide (DMSO) was purchased from Fisher Scientific (Leicestershire, UK). Betaine and hemin were purchased from Sigma Aldrich (St. Louis, MO, USA). The stock solution of hemin (10 μM) was prepared in DMSO and stored in the dark at 4 °C. Potassium chloride was purchased from Merck (Darmstadt, Germany); meanwhile, ABTS tablets were purchased from Amresco (Montreal, Canada). ABTS was prepared by dissolving 1 tablet of ABTS (10 mg) in 10 mL of 50 mM citric acid and 10 mL of 50 mM trisodium citrate from R&M (UK), with addition of 10 μL of 30% H_2_O_2_ from Nacalai Tesque (Kyoto, Japan). Absorbance measurements were performed using a Thermo Scientific™ Multiskan™ GO UV/Vis microplate spectrophotometer (MA, US). Meanwhile, a Nunc Maxisorp™ flat-bottom plate for absorbance reading was also purchased from Thermo Fisher Scientific (MA, US).

**Table 1:** Sequences of the synthetic oligonucleotides used. Bold and italic segments were designed to hybridize with the target-binding arms of Probes A and B, respectively. The underlined bases are G-forming sequences

Abbreviation	Strand	Sequences (5′–3′)
DENV-1TS	DENV-1 target strand	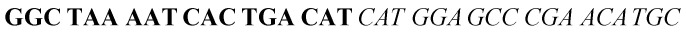
DENV-2TS	DENV-2 target strand	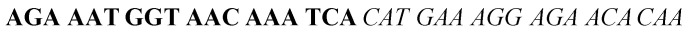
DENV-3TS	DENV-3 target strand	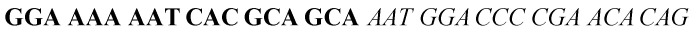
DENV-4TS	DENV-4 target strand	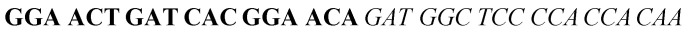
DENV-1PA	DENV-1 Probe A	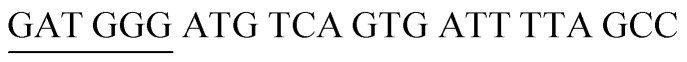
DENV-1PB	DENV-1 Probe B	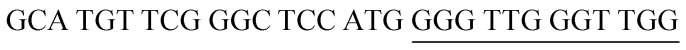
DENV-2PA	DENV-2 Probe A	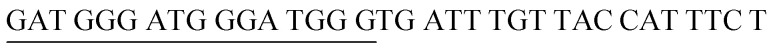
DENV-2PB	DENV-2 Probe B	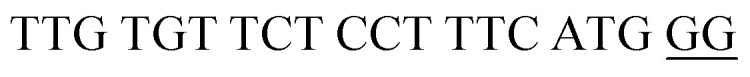
DENV-3PA	DENV-3 Probe A	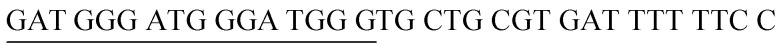
DENV-3PB	DENV-3 Probe B	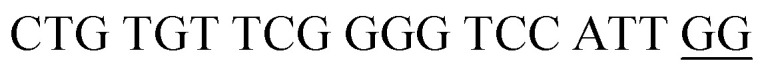
DENV-4PA	DENV-4 Probe A	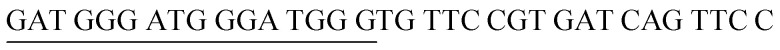
DENV-4PB	DENV-4 Probe B	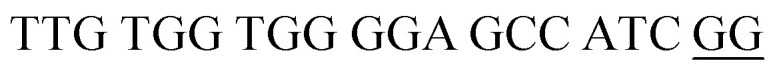

### Preparation of G4 structures

Samples were prepared in 4 sets (DENV-1-DENV-4). Firstly, 1 μM concentrations of target sequences with an equal concentration of Probe A and Probe B were mixed in a total reaction mixture of 15 μL containing a final concentration of 1 M betaine and 5% DMSO. The mixtures were then heated at 95 °C for 10 minutes to dissociate any secondary structures. The mixed solutions were gradually cooled to room temperature prior to adding 0.2 M potassium chloride and 2 μM hemin to a final volume of 25 μL for G4-hemin DNAzyme formation. The samples were then incubated for another 1 hour in the dark. The significance of adding the additives (betaine and DMSO) was also investigated.

In addition to testing the designated probes for each DENV serotype, the ability of a mixture of probes (multi-probe cocktail) to detect each of the DENV serotype strands was investigated. Herein, probes of each of the four different serotypes were mixed in an equimolar ratio. The preparation of this assay was similar to the preparation of the serotype-specific assay, except that the single set of probes was replaced with the multi-probe cocktail. To prepare the multi-probe cocktail, a total concentration of 1 μM Probe A (0.25 μM of each Probe A for DENV-1, DENV-2, DENV-3 and DENV-4) was mixed with 1 μM Probe B (0.25 μM of each Probe B for DENV-1, DENV-2, DENV-3 and DENV-4). The assay using the probe mixture was tested separately with DENV-1, DENV-2, DENV-3 and DENV-4 target strands. The concentration of each set of probes was deliberately minimized to limit the total concentration of the multi-probe cocktail and to equate the total probe concentration with the concentrations of the single-set probes.

### Colorimetric detection of DENV-1 – DENV-4 through the formation of G4-hemin DNAzyme

After 1 hour of incubation with potassium chloride and hemin, a total of 85 μL ABTS containing H_2_O_2_ was added to make up the total reaction volume to 110 μL, and the sample reactions were left in the dark. After 1 hour, the colour change of the ABTS-H_2_O_2_ reaction was observed and the absorbance was measured at the wavelength of 405 nm. The background reaction contained the same composition as the sample but without the target strand. All data were acquired in triplicate and are presented with the background absorption subtracted (unless stated otherwise), denoted as S-B.

### Selectivity of DENV probes

The cross-reactivity of each set of probes with different DENV serotypes was investigated to examine the selectivity of the probes for specific DENV serotypes. Oligonucleotides with sequences from two closely related flaviviruses (ZIKV: Zika virus; YFV: Yellow Fever virus) were synthesized and evaluated for their cross-reactivity with the DENV probes. The specificity of the multi-probe cocktail assay was also tested against these flaviviruses. The preparation steps were performed by replacing DENV target strands with ZIKV and YFV as target sequences. The sequences of ZIKV and YFV are listed in Table S2.†

### Sensitivity of the assay

The dependence of the signal absorbance on the target concentration was determined to analyse the sensitivity of the assay. In this approach, both the serotype-specific and cocktail assays were tested with various concentrations of target strands, namely 3.125 nM, 6.25 nM, 12.5 nM, 25 nM, 50 nM, 100 nM, 200 nM, 400 nM, 800 nM, 1600 nM, 3200 nM and 6400 nM. Next, the detection limits of the assays were determined by applying the equation of DL = 3*σ*_b_/slope.

## Results and discussion

### Principle of the biosensor

**Fig. 1:**
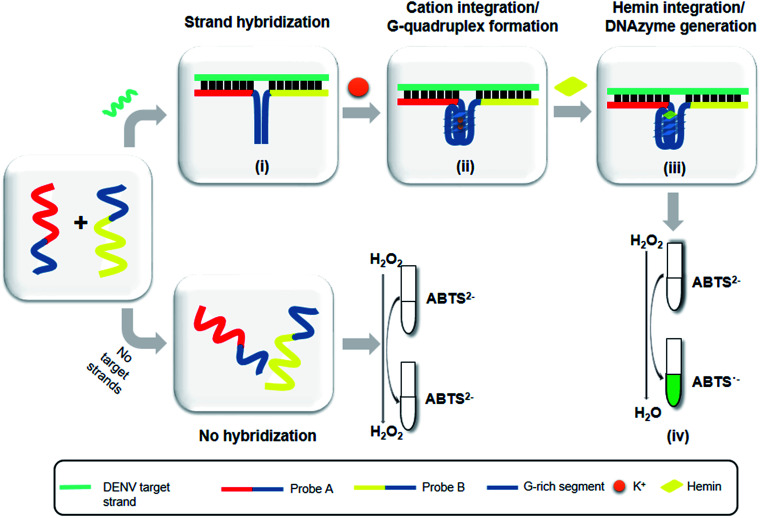
**-** Schematic of the principle of the DENV detection assay. (i) Target-probe strand hybridization. (ii) Integration of cations and formation of G4. (iii) Integration of hemin and generation of the G4-hemin DNAzyme. (iv) H_2_O_2_-mediated oxidation and colorimetric change of ABTS from colorless to a green product.

**Fig. 2:**
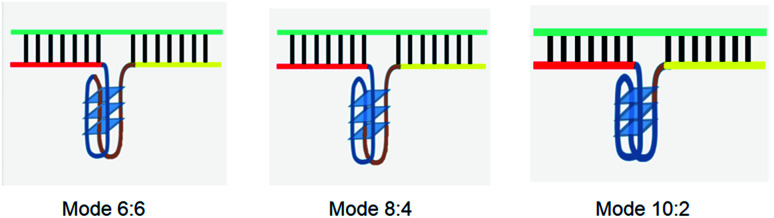
Splitting the G-quadruplex by three different modes.^12^ G-rich segments of Probe A are shown in blue; meanwhile the G-rich segments for Probe B are shown in brown. The mode ratios indicate the number of guanines located on each G-rich segment of each side of the probe.

The principle of this assay is depicted in Fig. 1. In this assay, G-rich sequences were split into two parts to act as binary probes for each DENV serotypes. As a binary probe, each G-rich segment was designed to anneal to the target-binding arm. The protruding G-rich ends were then brought together upon hybridization of the target-binding arms with the DENV target strand (DENV) to form a G4 structure in the middle with the aid of monovalent cations. Upon integration of hemin with the G4, the generation of the DNAzyme occurred. The ability of the formed DNAzyme to exhibit peroxidase-mimicking activity was used to catalyse the H_2_O_2_-mediated oxidation of colourless ABTS^2−^ to yield a green product, ABTS^−^. An illustration of the different split modes tested is shown in Fig. 2.

### Preparation of probe sequences

Variations within the DENV target genome arise from the mismatched sequences found throughout the genes of the different serotypes. This may cause a decrease in the specificity and sensitivity of assays, especially assays involving DENV isolates with different origins. Hence, DENV-specific probes were specifically designed to detect the RNA-dependent RNA polymerase (RdRp) regions of the DENV genome, a region that is well-conserved in the genome of flaviviruses.^39^ Although conserved, the RdRp gene still contains regions that are unique to each DENV serotype and to other closely related flaviviruses. On the other hand, the viral RNA polymerase is error-prone, which causes the DENV genome to accumulate mutations over time.^40^ These mutations result in genetic changes, which lead to the introduction of different/novel genotype(s). Therefore, being a region that is expectedly unchanged throughout the evolution, the RdRp region provides an ideal target gene for DNA sensing.

It was reported that split systems exhibit better selectivity due to the synergetic effects resulting from the conformational constraint and the binary approach.^41^ In this system, the frequently used total number of G bases used is twelve,^12^ and this number was preserved in our system. The G bases were initially distributed evenly (6 G bases each) between the two probes (*i.e.* 6 : 6). However, all four serotypes did not favour this split mode. The high background readings resulted in the failure of the system to detect the presence of DENV. It is possible that the 6 : 6 split mode is prone to self-assembling, which allows the strands to come together and generate a G4 in the presence of stabilizing ions. This assumption was supported by a previous study reporting that a high CD signal was generated when 6 : 6 was used for the split G4 test.^12^ To overcome this problem, different split modes were tested. It was previously reported that in a PPIX-integrated setup, the 4 : 8 mode provided the best readouts.^12^ Therefore, the 4 : 8 mode was adapted for DENV-1 sensing. The results showed an S-B that was high in comparison to that of the 6 : 6 mode and was sufficient for effective target detection; hence, this mode was used as the probe split mode for DENV-1. Because DENV-1 shares a relatively high similarity (66.67%) with DENV-3, the same mode (4 : 8) was tested on DENV-3. However, although the signal was considerably high, the high background produced greatly affected the sensitivity of the assay for DENV-3. Therefore, a different split mode at 10 : 2 with a shorter G-segment was tested on DENV-3. The results showed that the S-B obtained by applying 10 : 2 mode was higher than the result shown when applying 4 : 8 mode. The same mode (10 : 2) was then evaluated on DENV-2 and DENV-4, as they share sequence similarities in the same range as DENV-3 of 58.33% and 69.44%, respectively. The results demonstrated that the backgrounds were significantly lower relative to the signal readouts.

**Fig. 3:**
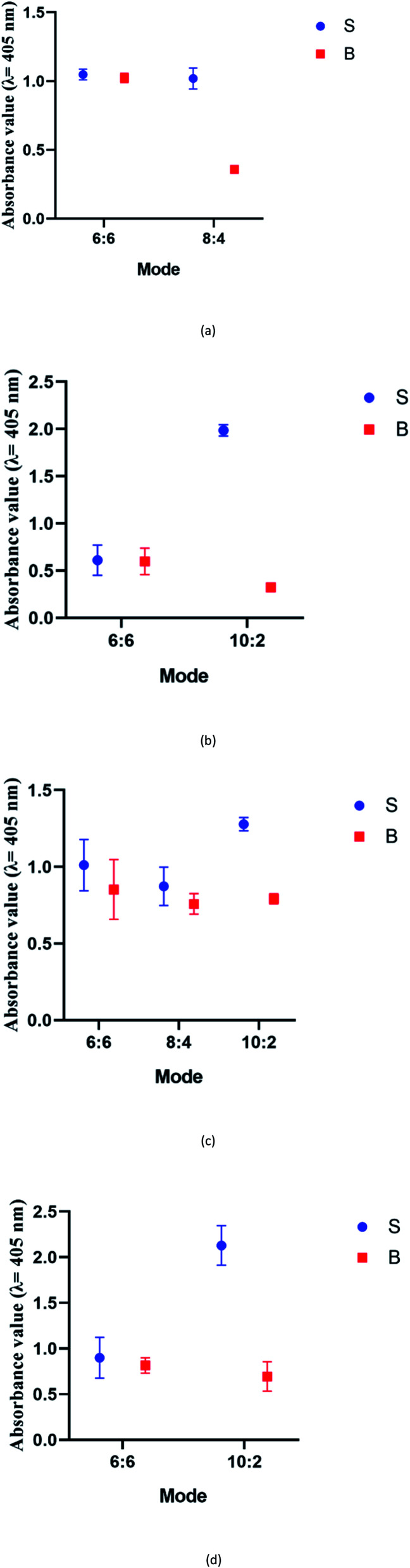
Efficacy of different modes for (a) DENV-1, (b) DENV-2, (c) DENV-3, and (d) DENV-4 based on the signal and background readings.

We suspect that the effectiveness of this mode may be due to the role of the two G bases in the short segments in G4 formation with the neighbouring ten G bases. It is likely that the ten-G strand is distributed between the G-tracts at a ratio of 3 : 3 : 3 : 1. The two protruding G on the other strand, which is brought in close proximity due to the target hybridization, could potentially be attracted to the strand, enabling complete G4 formation. In the absence of the two G, the ten-G stretch was unable to form a complete stable quadruplex structure; this inhibited DNAzyme formation, resulting in low background signals. Therefore, the optimum designs were the 4 : 8 split mode for DENV-1 and the 10 : 2 split mode for DENV-2, DENV-3, and DENV-4. The signal (S) and background (B) readings obtained from each tested mode are presented in Fig. 3. Table 1 shows the sequences of the targets and probes used for the assay. The oligonucleotide sequences used for split mode optimization are listed in Table S1.†

### The formation of G4-hemin DNAzyme for the detection of DENV serotypes

**Fig. 4:**
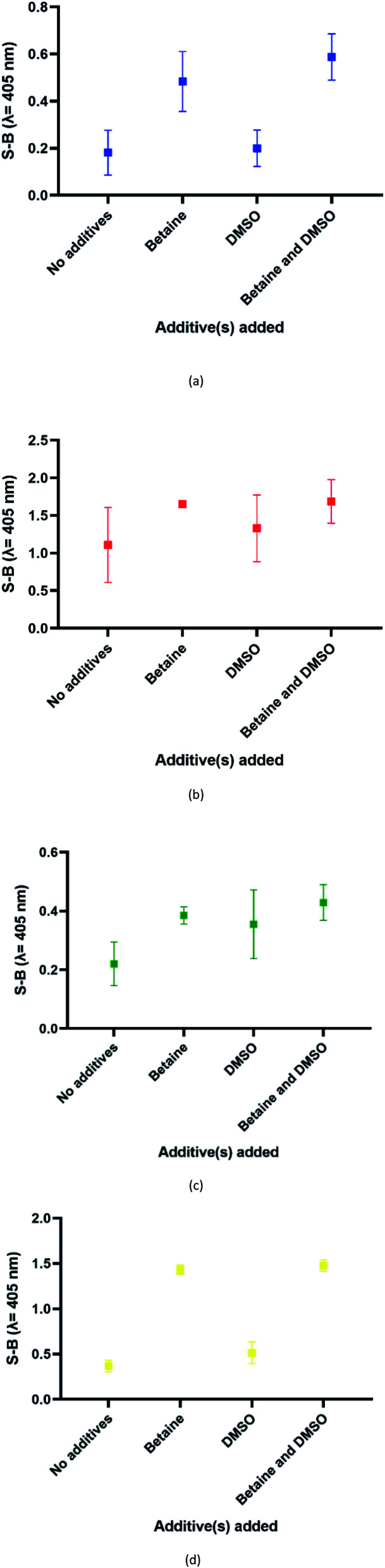
Effects of addition of different additive(s) on the efficacy of the (a) DENV-1, (b) DENV-2, (c) DENV-3 and (d) DENV-4 assays (without additives, betaine only, DMSO only and betaine and DMSO).

In this assay, potassium ion (K^+^) was chosen over other commonly used cations such as sodium ions to promote the formation of G4 due to its reported biological relevance.^42–44^ The proposed split G4 formation was highly dependent on the three-strand complex hybridization. Even so, some oligonucleotide strands may be inefficient for hybridization due to the presence of intramolecular interactions. This may limit the sensitivity of the assay because the regions of interest may be affected. In our approach, both the target and probe strands were denatured at 95 °C with the aid of DMSO and betaine before the addition of potassium ion for G4 formation. Fig. 4 shows the influences of different additives on the generated S-B. Independently, betaine increased the S-B for all four serotype assays, with the highest increments in DENV-1 (Fig. 4(a)) and DENV-4 (Fig. 4(d)). However, DMSO did not exhibit a significant increase of the S-B readout. A much higher S-B was obtained when betaine was used in combination with DMSO. This demonstrates the combinatorial effect of DMSO and betaine in strand hybridization and its implications in G4 formation. This was likely due to the nature of the additives, which function to disrupt secondary structure formation and eliminate its adverse effects to enhance the hybridization of the probes to the target strands. Betaine functions as a molecular barrier to lower the association rate constant, *K*_1_, for hybridization and lowers the hybridization efficiency.^45^ These additives have been shown in the past to enhance amplification of long PCR products, which reduces the likelihood of intramolecular stem loop formation by single-stranded templates during amplification.^46^

**Fig. 5:**
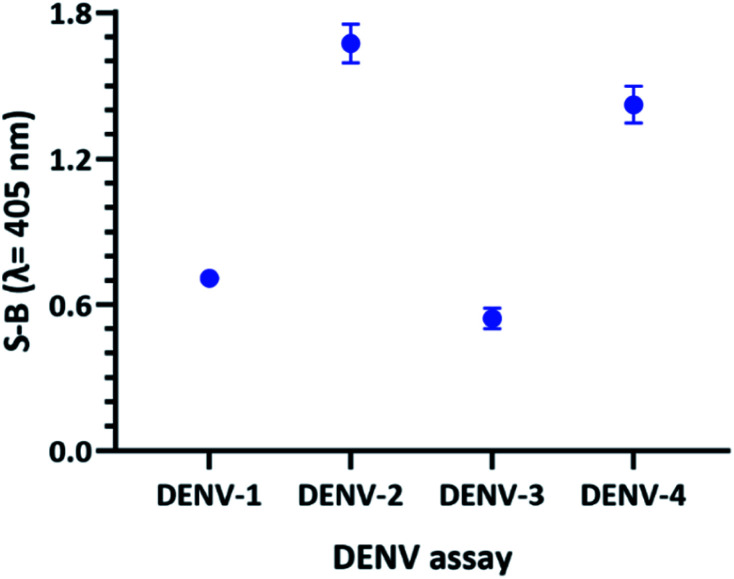
Absorbance readings generated by the DENV-1–DENV-4 assays, reflecting the efficacy of the DENV detection assays.

To obtain a reliable assay, the catalytic activities of the four sets of probes were investigated and compared under the same conditions. Both betaine and DMSO were added in this work to enhance the strand hybridization. Once G4 formation occurs in the presence of potassium ions, the integration of the hemin molecule into the G4 complex will result in the formation of a DNAzyme with peroxidase-like activity. Hemin-catalysed H_2_O_2_-mediated oxidation of ABTS^2−^ to ABTS^−^ will result in a visible colour change. Fig. 5 shows the efficacy of the designed probes to identify the specific DENV serotypes *via* the presence of catalytic activity, which generates absorbance readouts. The absorbance readouts for the assays of all the serotypes indicate detection of the target strands, which represents successful target hybridization and G4 formation.

### Specificity of the designated probes for their respective DENV target strands

**Fig. 6:**
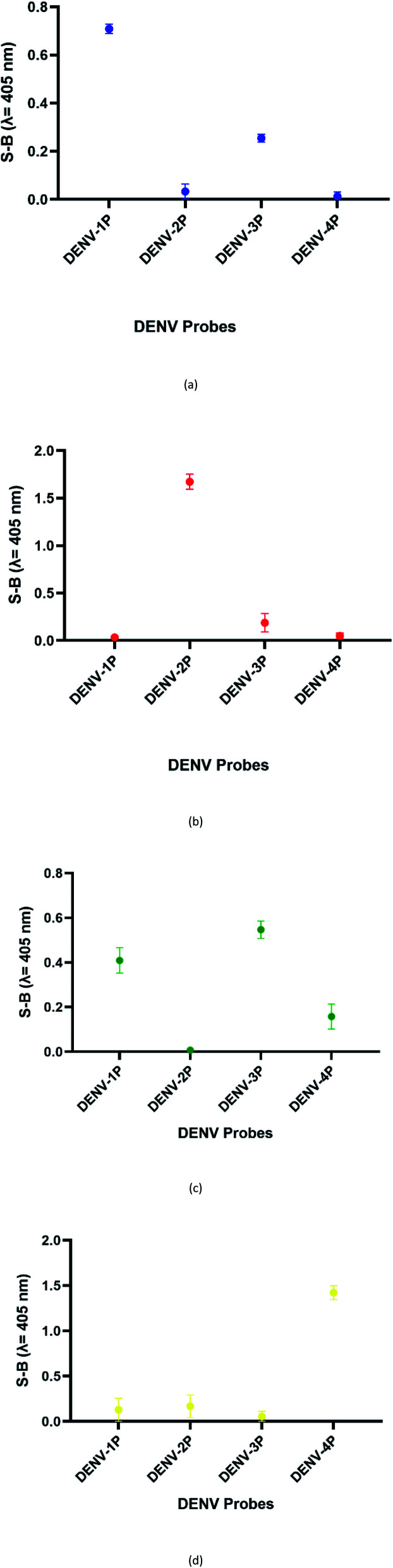
Absorbance readings generated by the (a) DENV-1, (b) DENV-2, (c) DENV-3 and (d) DENV-4 assays in the presence of target strands of the other serotypes, reflecting the specificity of the designated DENV probes.

DENV shares some sequence similarities within the DENV serotypes, which may cause the designated serotype probes to cross-react with other serotypes. The homologous bases among DENV target strands are illustrated in Fig. S1.† As shown in Fig. 6, all probes were specific towards their respective targets. However, DENV-1P and DENV-3P exhibited low reactivity with DENV-3TS and DENV-1TS, respectively, which may be caused by the high sequence similarity of DENV-1TS and DENV-3TS used in this assay. A segment of the DENV-1P target-binding arm could potentially anneal to DENV-3TS due to the presence of a long continuous complementary base region on both DENV-1PA and DENV-1PB. Therefore, the two G-rich segments could react with each other. However, the gaps generated due to the presence of non-complementary bases in between the complementary regions could aid the formation of the G4 structure with low integrity, resulting in the generation of low catalytic activity. The same explanation could be applied to the reaction between DENV-1TS and DENV-3P. The latter possibility has been proven true by a group investigating the relationship of the base number gap with the integrity of the G4 structure.^5^ However, DENV-1P and DENV-3P showed greater selectivity towards their respective targets. This enabled DENV-1TS and DENV-3TS to be discriminated, provided that each set of DENV probes were applied to the target samples. On the other hand, DENV-2P and DENV-4P showed excellent specificity toward DENV-2TS and DENV-4TS, respectively.

Although the DENV-3TS and DENV-4TS strands used in this assay shared the highest sequence similarity, DENV-3P showed no cross-reactivity with DENV-4TS, and DENV-4P did not show any cross-reactivity with DENV-3TS. Referring to the oligonucleotide sequences used in this assay, there was only one long continuous complementary base region for the binding of DENV-3TS and DENV-4P as well as the binding of DENV-4TS and DENV-3P to occur. These complementary bases were located on only one side of the probes, and the G4 complex could not be formed with only one side of the binary probes.

### Cross-reactivity of the DENV probes with other flaviviruses

**Fig. 7:**
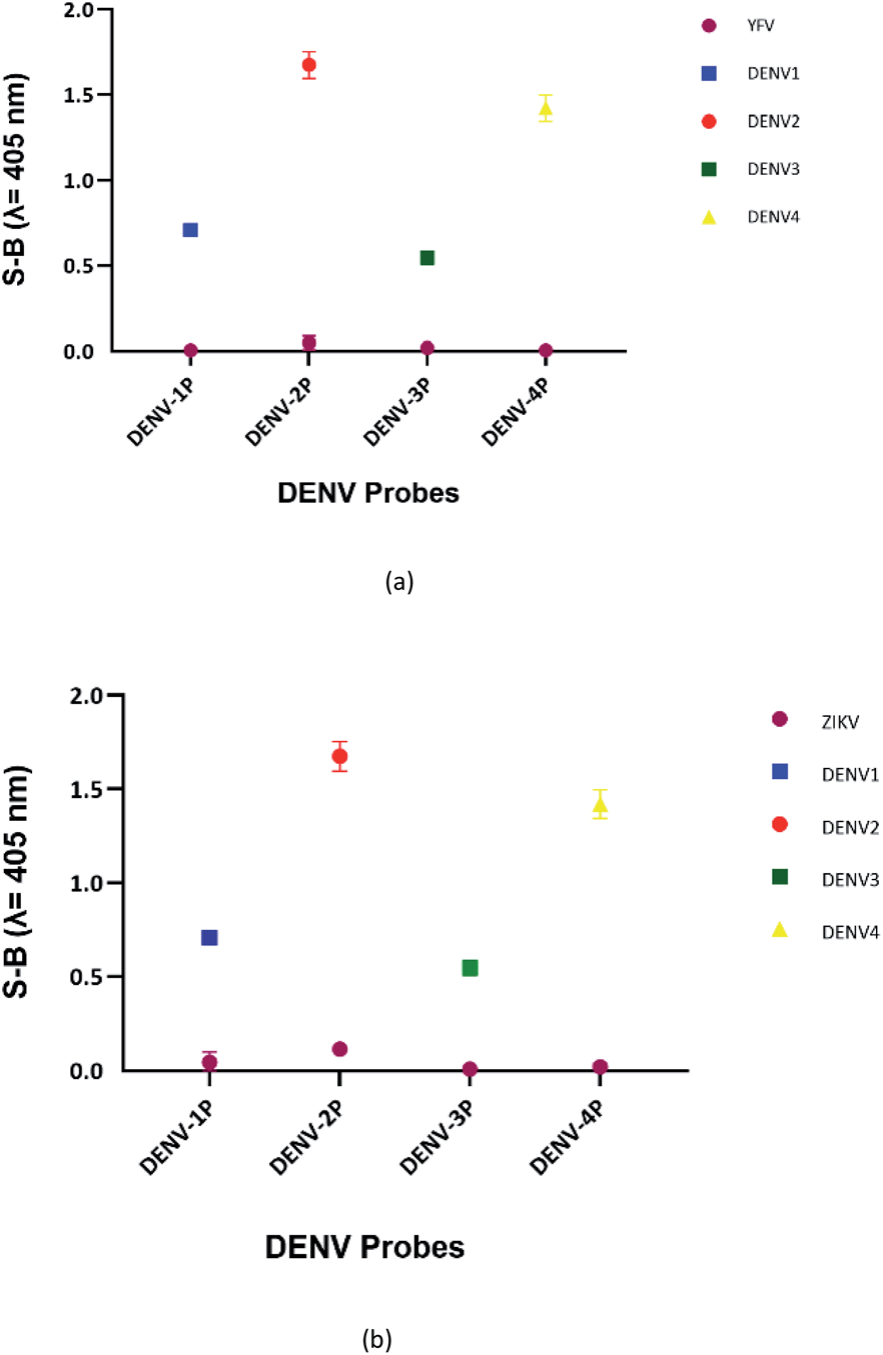
Absorbance readings generated by each set of assays in the presence of target strands of (a) ZIKV and (b) YFV, showing the specificity of the DENV probes.

The sensitivity of this assay was further verified by analysing the cross-reactivity of its probes with other flaviviruses. Two closely related flaviviruses, Zika virus (ZIKV) and Yellow Fever virus (YFV), were tested. No distinct colour changes were observed in the target region of the other flaviviruses, indicating that the assay was very specific to DENV (see Fig. 7).

### Efficacy of the multi-probe cocktail for DENV detection

**Fig. 8:**
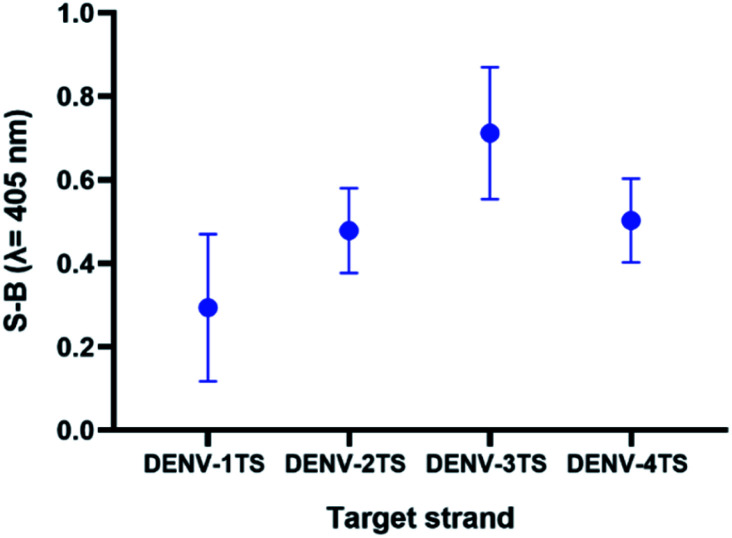
Efficacy of DENV-1TS, DENV-2TS, DENV-3TS and DENV-4TS detection using the multi-probe cocktail assay.

As the possibility of two serotypes being present at the same time is low, we decided to evaluate the performance of a multi-probe cocktail system for detection. Because the RdRp regions of each DENV serotype share high sequence similarity, it was suggested that a specific DENV strand might react minimally with the probes of the other serotypes. Fig. 8 shows the readings of the assay using a cocktail of probes in the presence of each serotype target strand. The highest reading was shown by the reaction of the multi-probe cocktail with DENV-3TS, followed by DENV-4TS, DENV-2TS and DENV-1TS. The difference in the readings is most likely due to the differences in the percentage of sequence complementary of the DENV target strand with each of the probe sets. Among all of the target strands, DENV-3TS exhibited the highest percentage of sequence complementary with the rest of the probe sets (69.44% with DENV-4P, 66.67% with DENV-1P and 58.33% with DENV-2P). Hence, it is possible that DENV-3TS reacted not only with its own probes but with the other probes in the cocktail. In general, the S-B generated by the cocktail assay was lower than those of the single-set-probe detection assays in the previous section. This can be explained by the presence of higher probe competition for hybridization with the target strands between the multiple probes. Another possibility is that the short complementary segments provide weak hybridization strength, thus allowing the strands to dissociate easily.^47^

### Cross-reactivity of the multi-probe cocktail with other flaviviruses

**Fig. 9:**
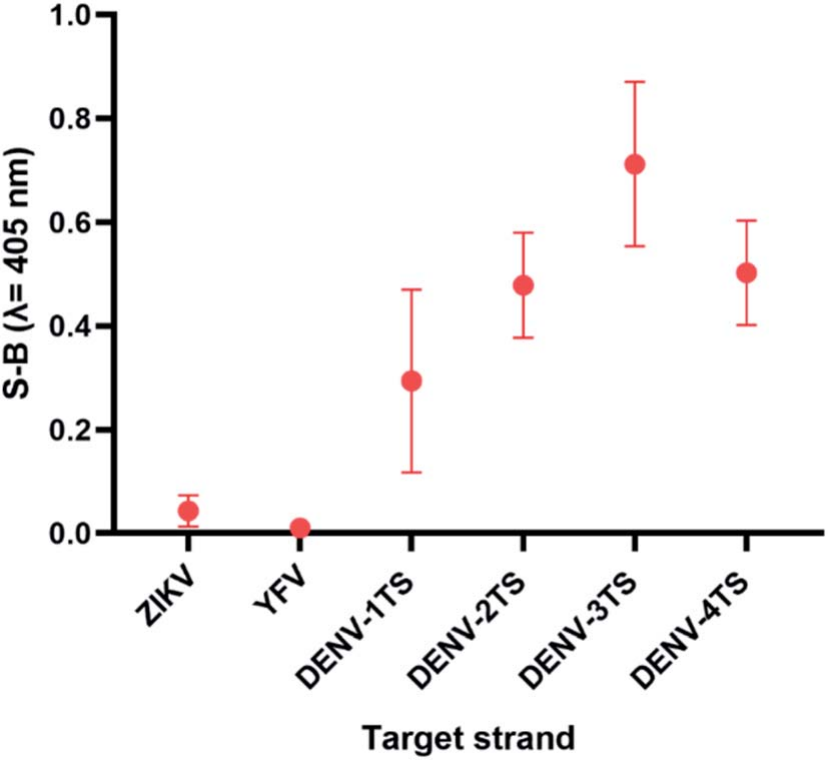
Selectivity of the multi-probe cocktail for each DENV target strand.

Using the multi-probe cocktail assay, no apparent S-B was generated when ZIKV and YFV were respectively added (see Fig. 9). This indicates the specificity of the probes for DENV detection, with no cross-reaction with ZIKV or YFV. The S-Bs of the assay in the presence of each DENV serotype target strand were also compared. The reaction of this assay with the flaviviruses can be explained by the low number of complementary bases and the short length of the complementary segments. Weak strand hybridization or the inability of strands to hybridize could cause the failure of the whole DNAzyme generation pathway.

### Sensitivity of the assay

Under the same conditions, the relationship between the concentration of target DNA and the S-B was also evaluated. The proposed system was challenged with various target concentrations, namely 3.125 nM, 6.25 nM, 12.5 nM, 25 nM, 50 nM, 100 nM, 200 nM, 400 nM, 800 nM, 1600 nM, 3200 nM and 6400 nM. In this experiment, the relationships of the target strand concentrations with the S-B of both assays (serotype-specific assay and multi-probe cocktail assay) were evaluated. It was evident that as the target concentrations increased, the catalytic activity of the assays also increased up to a certain point (see Tables 2 and 3). However, we noticed that the catalytic activity decreased when the target strands were present in excess.

**Table 2:** Absorbance readings generated by the serotype-specific assay in the presence of various concentrations of the respective DENV target strands

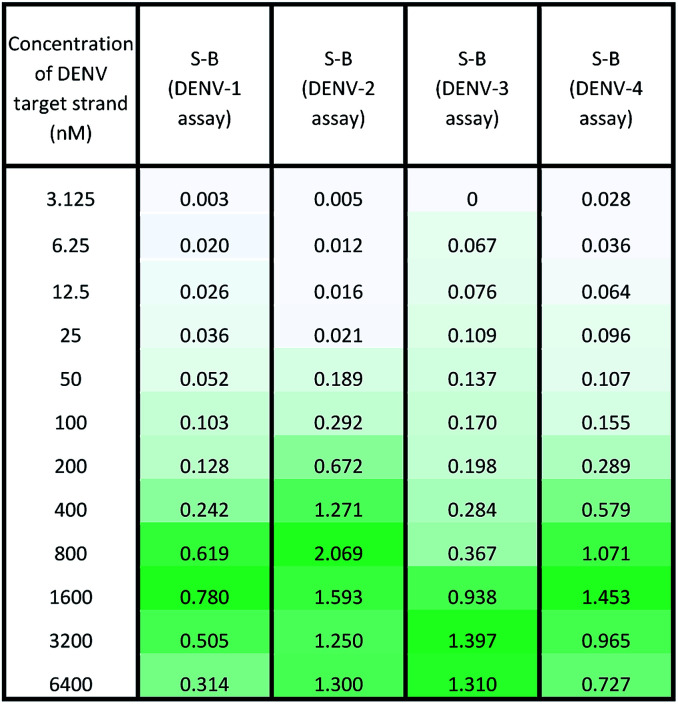

**Table 3:** Absorbance readings generated by the multi-probe cocktail assay in the presence of various concentrations of different DENV serotype target strands

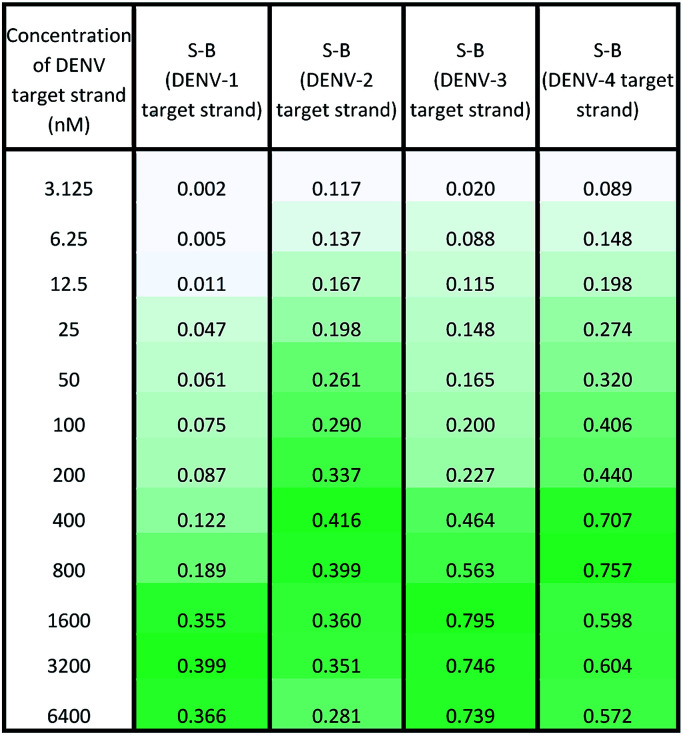

In the serotype-specific assays, this was revealed by the drop in S-B when the concentrations of the target strands were over 1600 nM, 1000 nM, 3200 nM and 1600 nM, respectively, for the DENV-1, DENV-2, DENV-3 and DENV-4 assays. For the cocktail assay, a drop in the S-B could be observed when the target concentrations were beyond 3200 nM, 400 nM, 1600 nM and 800 nM, respectively, for each serotype. It is suggested that when excess target strands are present in comparison to the probe strands, each of the two probes can predictably hybridize with a single target strand independently, resulting in failure to bring the two probes in proximity to form a G4 structure. This same phenomenon has been observed in previous studies, where an obvious decrease in catalytic activity occurred with the use of excess target strands.^48,49^ In the described studies, it was presumed that the G-rich protruding parts entirely hybridized with the target strands, causing the G4 structure to dissociate.

The catalytic activity of the assays was greatly enhanced with the addition of low levels of target strands. By applying the equation of DL = 3*σ*_b_/slope (where *σ*_b_ = the standard deviation of the blank), the detection limits of the serotype-specific assays were determined to range from 4.9 nM to 9.3 nM, *i.e.* DENV-1: 8.8 nM, DENV-2: 4.9 nM, DENV-3: 9.3 nM and DENV-4: 5.1 nM. The difference in the detection limits obtained may be influenced by the different hybridization strengths of the three-strand complex. Meanwhile, the limits of detection for the cocktail assay were identified to be 36.7 nM, 42.6 nM, 35.2 nM and 37.1 nM for the addition of DENV-1TS, DENV-2TS, DENV-3TS and DENV-4TS, respectively. Note that the serotype-specific assays and cocktail assay exhibited high differences in their detection limits. The cocktail assay possessed higher detection limits; this is most likely associated with the low concentration of each set of designated probes used in this assay. As mentioned, the low concentrations were used to reduce the total concentration of the mixed probes and to equilibrate the total concentration of the probes with the final concentration of the serotype-specific assay. However, both types of assay offered detection limits in the nanomolar range. Table 4 shows the comparative advantages and disadvantages of some other widely used methods for DENV serotyping. The detection limit of the assay is higher than 3 nM, which may be challenging for detection of the number of virus particles in actual samples. Due to this apparent shortcoming, direct detection of virus particles would be inefficient. However, the developed system can be used in combination with other amplification methods, such as isothermal amplification, or PCR amplification methods such as those listed in Table 4, where the approach can be adapted as a colorimetric reporting system.

**Table 4:** Comparative advantages and disadvantages of the split G4 DNAzyme-based assay with DNA microarrays, real time reverse-transcription PCR (qRT-PCR), reverse transcription loop-mediated isothermal amplification (RT-LAMP) and semi-quantitative nested PCR

DENV serotyping assay	Split G4 DNAzyme-based assay^50^	DNA microarray^51^	qRT-PCR^52,53^	RT-LAMP^54^	Semi-quantitative nested PCR^55^
Advantages	Inexpensive	Can detect dual infection of two different DENV serotypes	Able to do quantitative measurements	Able to do quantitative measurements	Involves negation of improper primer binding; hence, non-specific detection could be reduced
	Easy to perform and portable		Lower contamination rates due to closed tube operation	Able to do naked-eye visualization	
	Amplification-free, lowering risk of target strand contamination		Involves software-driven operation and hence can be applied in high-throughput analysis		
	Enables naked-eye visualization				
Disadvantages	Unable to perform measurements of target concentrations	Requires specialized and expensive instruments	Requires trained personnel	Complicated design of primers (requires six primers)	Contamination of amplicon products may occur because targets are detected using two sets of primers for a double process of amplification
		Restricted to laboratories with good financial support	Expensive detection equipment and consumables	HPLC purification is needed for two long primers	
		Requires trained personnel	Uneconomical for an average laboratory		
			Requires fluorescent probes		
Requirement of secondary method (agarose gel electrophoresis analysis/ethidium bromide or SYBR green integration)	No	No	No	Optional	Yes
Requirement of thermal cycler/sophisticated instruments	No	Yes	Yes	No	Yes

## Conclusions

A simple label-free colorimetric system for DENV serotyping and detection was successfully established using a split G4 configuration. The enhanced hybridization using betaine and DMSO between the target DNA and the recognition region of the serotype-specific split probes could enhance the generation of a functional G4 DNAzyme. This was used to provide a better positive readout signal to achieve the desired colorimetric characteristics. Although nucleic acid amplification-based approaches are reported to be highly sensitive and specific for DENV serotyping, the use of a split G4 formation-based system is an advantage in that it involves a very simple operational method. It must be taken into consideration that when a method is to be employed for diagnosis, different laboratories have different accessibilities to the components of the reactions. Despite being PCR-free and requiring no chemical modification, the developed DENV serotyping assay possesses detection limits of 4.9–9.3 nM and 35.2–42.6 nM for the serotype-specific and multi-probe cocktail DENV detection assays, respectively. This may hamper the application of this approach for direct virus detection; however, it can be used as an alternative reporter system with other DNA amplification methods. Because the sequences of the two G-rich segments can be flexibly adjusted according to the target DNA, this platform could be widely adapted for various nucleic acid detection assays or serotyping of other viruses. A major shortcoming of the approach is the inability to test the assay with actual virus mRNA; we envision the expansion of the scope of our assay to determine its clinical applicability for biological samples. To further validate the assay, field testing in various DENV endemic areas is also required. The ability of a split G4 configuration to yield stable functional DNAzymes provides another new dimension for DNA-based sensing using G4 structures.

## Funding

T. S. L. acknowledges the Malaysian Ministry of Higher Education for financial support through the Fundamental Research Grant Scheme (203/CIPPM/6711658 - Reference Code: FRGS/1/2018/STG05/USM/02/2). J. I. would like to acknowledge support from Sarawak Foundation Tun Taib Scholarship.

## Conflicts of interest

There are no conflicts of interest.

## Supplementary Material

RA-010-D0RA05439A-s001
