# Supplementary material for: An intermolecular-split G-quadruplex DNAzyme sensor for dengue virus detection
Source: RSC Adv. 2020 Sep 7;10(55):33040–51. doi: 10.1039/d0ra05439a (PMC9056686; doi:10.1039/d0ra05439a)
Supplement: RA-010-D0RA05439A-s001 [file RA-010-D0RA05439A-s001.pdf]

Electronic Supplementary Material (ESI) for RSC Advances.
This journal is © The Royal Society of Chemistry 2020

Supplementary Materials

Table S1 - Oligonucleotide sequences used for split mode optimization. The underline bases are G4-forming segments.

Oligonucleotide	Sequence (5'-3')
DENV1 Probe A (6 guanine)	GGG AT GGG ATG TCA GTG ATT TTA GCC
DENV1 Probe B (6 guanine)	GCA TGT TCG GGC TCC ATG GGG TT GGG
DENV2 Probe A (6 guanine)	GGG AT GGG TGA TTT GTT ACC ATT TCT
DENV2 Probe B (6 guanine)	TTG TGT TCT CCT TTC ATG GGG TT GGG
DENV3 Probe A (6 guanine)	GGG AT GGG TGC TGC GTG ATT TTT TCC
DENV3 Probe B (6 guanine)	CTG TGT TCG GGG TCC ATT GGG TT GGG
DENV3 Probe A (4 guanine)	G AT GGG TGC TGC GTG ATT TTT TCC
DENV3 Probe B (8 guanine)	CTG TGT TCG GGG TCC ATT GGG TT GGG TT GG
DENV4 Probe A (6 guanine)	GGG AT GGG TGT TCC GTG ATC AGT TCC
DENV4 Probe B (6 guanine)	TTG TGG TGG GGA GCC ATC GGG TT GGG

Table S2 - Oligonucleotide sequences of other flaviviruses used in cross-reactivity test.

Oligonucleotide	Sequence (5'-3')
Zika virus	AGC CTT GAT TAC TTA CCA AAT GGA TGA AGG GCA CAG
Yellow fever virus	ACA GGA GAT CTT GAA CTA CAT GAG CCC ACA TCA CAA

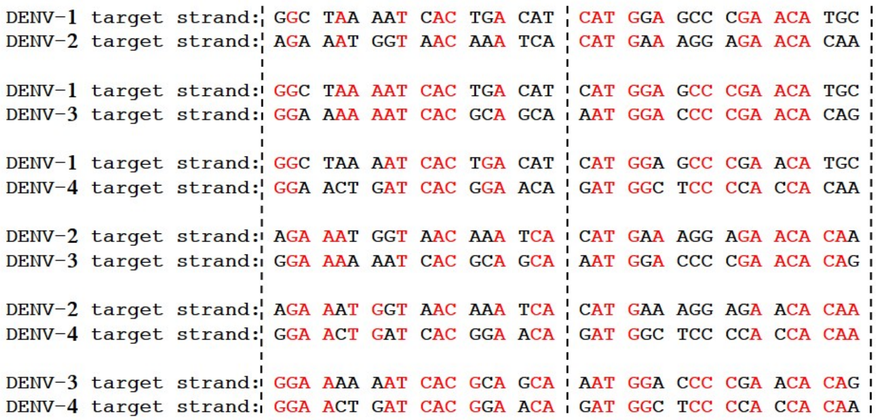

(a) (b)

Figure S1 - Red bases show the homologous sequences among DENV serotypes. (a) Region annealing to Probe A and (b) Region annealing to Probe B.
